# New Insights into the Structure and Assembly of Bacteriophage P1

**DOI:** 10.3390/v14040678

**Published:** 2022-03-25

**Authors:** Miguel F. Gonzales, Denish K. Piya, Brian Koehler, Kailun Zhang, Zihao Yu, Lanying Zeng, Jason J. Gill

**Affiliations:** 1 Center for Phage Technology, Texas A&M University, 2128 TAMU, College Station, TX 77843, USA; mikefg@tamu.edu (M.F.G.); denish.piya@gmail.com (D.K.P.); kailun@wustl.edu (K.Z.); zihao.yu@tamu.edu (Z.Y.); lzeng@tamu.edu (L.Z.); 2 Interdisciplinary Program in Genetics, Texas A&M University, 2128 TAMU, College Station, TX 77843, USA; 3 Department of Biochemistry and Biophysics, Texas A&M University, 2128 TAMU, College Station, TX 77843, USA; bkoehler1996@gmail.com; 4 Department of Animal Science, Texas A&M University, 2471 TAMU, College Station, TX 77843, USA

**Keywords:** bacteriophage P1, bacteriophage structure, proteomics, capsid targeting sequence, defense against restriction, transmission electron microscopy

## Abstract

Bacteriophage P1 is the premier transducing phage of *E. coli*. Despite its prominence in advancing *E. coli* genetics, modern molecular techniques have not been applied to thoroughly understand P1 structure. Here, we report the proteome of the P1 virion as determined by liquid chromatography tandem mass-spectrometry. Additionally, a library of single-gene knockouts identified the following five previously unknown essential genes: *pmgA*, *pmgB*, *pmgC*, *pmgG*, and *pmgR*. In addition, proteolytic processing of the major capsid protein is a known feature of P1 morphogenesis, and we identified the processing site by N-terminal sequencing to be between E120 and S121, producing a 448-residue, 49.3 kDa mature peptide. Furthermore, the P1 defense against restriction (Dar) system consists of six known proteins that are incorporated into the virion during morphogenesis. The largest of these, DarB, is a 250 kDa protein that is believed to translocate into the cell during infection. DarB deletions indicated the presence of an N-terminal packaging signal, and the N-terminal 30 residues of DarB are shown to be sufficient for directing a heterologous reporter protein to the capsid. Taken together, the data expand on essential structural P1 proteins as well as introduces P1 as a nanomachine for cellular delivery.

## 1. Introduction

Enterobacterial phage P1 is one of the earliest described temperate phages [1]. P1 is a well-known workhorse of molecular genetics due to its ability to transduce host DNA at high frequency [2] and across diverse bacterial species [3]. Further, P1 has played a role in the study of bacterial restriction [4] leading to the identification of the multicomponent defense against the restriction (Dar) system of P1 [5,6]. Phage P1 is now recognized as a member of a larger group of temperate phages that maintain themselves as plasmids and are associated with antibiotic resistance determinants [7] and virulence factors [8] in *E. coli*. Phage P1 and its relatives appear to be a unique phylogenetic group with no clear relationships to other phages and are currently designated by the ICTV as the unclassified phage genus *Punavirus*.

The P1 genome is understood to encode 119 genes, with 14 annotated structural proteins based mainly on an analysis of structural defects in P1 amber mutants [9,10]. In addition to these essential structural proteins, P1 also encodes the multi-component Dar antirestriction system which is composed of 6 proteins, two of which affect capsid morphogenesis [6]. However, a number of genes in P1′s 94.5 kb genome have functions that are vaguely, if at all, defined. For instance, 18 genes are classified as ‘putative’, and 22 of the 119 predicted genes of bacteriophage P1 have no known function. Thus, it is still relevant to apply modern molecular methods to understand the structure of P1 to the level of other paradigm phages such as T4 and lambda [11,12].

Here, we present a complete proteomic profile of the P1 virion and identify essential structural proteins by a combination of whole virion mass spectrometry, transmission electron microscopy of P1 single-gene knockouts, and bioinformatic analyses. Additionally, we identify the peptide maturation cleavage site of the P1 major capsid protein, elucidate the capsid localization signal of the 250 kDa protein DarB, and show that this signal can target a heterologous protein to the phage capsid.

## 2. Materials and Methods

### 2.1. Bacterial Strains and Phages

The bacterial strains and the parental phage P1CM*clr*100 (hereafter referred to as P1) used in this study were obtained from the Coli Genetic Stock Center, Yale University, or from our previous studies [6]. The full list of strains is shown in supplementary Table S1 and handled as previously described [6]. Unless otherwise noted, *E. coli* strains were cultured in LB (10 g/L Bacto Tryptone (BD Biosciences, Franklin Lakes, NJ, USA), 5 g/L Bacto yeast extract (BD Biosciences), 10 g/L NaCl (Avantor, Radnor, PA, USA)) or LB agar (LB amended with 15 g/L Bacto agar) and incubated at 37 °C. P1 lysogens were cultured and maintained at 30 °C on LB amended with 10 μg/mL chloramphenicol (LB Cm-R) or 10 μg/mL chloramphenicol plus 30 μg/mL kanamycin (LB Cm-R Kan-R).

### 2.2. Production of Phage Lysates

Phage lysates were produced by thermal induction of P1 lysogens as described previously [6]. Briefly, an *E. coli* P1 lysogen was grown at 30 °C in LB Cm-R to an OD_550_ between 0.5 and 0.6. Next, the P1 lysogen was thermally induced by shifting the culture to 42 °C [5] until the OD_550_ fell below 0.2. Then, chloroform was added (0.1% *v*/*v*) to the lysate. Finally, the lysate was centrifuged at 10,000× *g* for 30 min, and the supernatant was stored at 4 °C until further use.

### 2.3. Transmission Electron Microscopy

Phages were stained with 2% uranyl acetate and imaged in a JEOL 1200 EX transmission microscope under 100 kV accelerating voltage as previously described [13,14].

### 2.4. pBAD24g Plasmid Construction

The gentamicin resistance cassette was PCR amplified from plasmid pMB838 with primers designed to have AatII and SacI restriction sites on the 5′ and 3′ end of forward and reverse primers, respectively. Similarly, the backbone of plasmid pBAD24 was PCR amplified with primers designed to have SacI and AatII restriction sites on the 5′ and 3′ end of forward and reverse primers, respectively. All PCR reactions were conducted using Phusion Hi-Fidelity PCR Master Mix (New England Biolabs, Ipswich, MA, USA) following the manufacturer’s recommended protocol. The gentamicin resistance cassette was ligated into pBAD24 backbone using standard molecular biology techniques. P1 genes *pacA*, *pmgA, pmgB, pmgC, pmgG* and *pmgR* were cloned into pBAD24g (g for gentamicin resistance). These genes were PCR amplified using primers with XbaI and HindIII restriction sites on 5′ end and were ligated into the respective restriction sites in pBAD24g following standard molecular biology techniques.

### 2.5. Generation of Single-Gene Knockout Mutants

Lambda Red recombinase-mediated homologous recombination was used to generate isogenic single-gene knock-out P1 mutants as described previously [6,15]. Briefly, P1 was lysogenized into BW25113 (pKD46), and colonies resistant to both chloramphenicol and ampicillin were selected. BW25113 (pKD46) lysogenized with P1 was induced with arabinose, made electrocompetent, and transformed with linear PCR products containing a *kan* cassette and FRT sites with flanking regions homologous to the targeted insertion site as described previously [6]. The recombinant P1 phages were lysogenized into *E. coli* strain MG1655 as described previously [6] for maintenance.

### 2.6. Generation of DarB-tetR-mCherry Capsid Localization Sequence Fusion Protein

Synthetic DNA fragments that encompassed regions of plasmid pBAD24 from 50 bp upstream of the NheI restriction site to 50 bp downstream of the HindIII restriction site, with some modifications, were used. The ribosome binding site was modified to “aggaggt”, followed by eight arbitrary nucleotides. The nucleotides encoding the N-terminal 9 or 30 residues of DarB (including the first residue Met) were added downstream of the arbitrary nucleotides. The pBAD24 multiple cloning sites from EcoRI to HindIII were added downstream of the *darB* specific nucleotides. Both synthetic DNA fragments were PCR amplified and ligated into NheI and HindIII restriction sites of pBAD24 using standard molecular biology techniques. TetR-mCherry [16] was PCR amplified with primers designed to have EcoRI and KpnI restriction sites on the 5′ end and ligated into pBAD24 to generate pBAD24_N30-tetR-mCherry, pBAD24_N9-tetR-mCherry, and pBAD_tetR-mCherry.

### 2.7. Generation of N-terminal DarB Mutants

For constructing plasmid expressing DarB with N-terminal truncations, the start codon ATG was included in the forward primers. The 5′ end of forward and reverse primers were designed to include XbaI and HindIII restriction sites. The amplified PCR product was ligated into XbaI and HindIII sites in pBAD24 following standard molecular biology techniques.

### 2.8. Generation of Internal DarB Truncation Mutants

Internal truncation mutants of DarB were developed using Q5^®^ Site-Directed Mutagenesis Kit (New England Biolabs). Primers were designed to truncate the N-terminal sequence and the amplicon was cloned into pBAD24 using the XbaI and HindII sites using standard molecular biology techniques following the manufactures suggestions. The internal truncations were generated using the Q5^®^ Site-Directed Mutagenesis Kit (New England Biolabs) using pBAD24_*darB* as a template. The amplicons were gel purified and used to transform 5-alpha competent *E. coli* (NEB) by heat shock and plated on selective media.

### 2.9. Efficiency of Plating (EOP) Assays

All phages used in EOP assays were lysogenized into *E. coli* WA921 and phages produced by induction as previously described [6]. EOP assays were conducted following previously established protocol, with minor modifications [6]. P1 Phages were serially diluted 10-fold in SM buffer (100 mM NaCl, 8 mM MgSO_4_, 50 mM Tris-HCl pH 7.5) and 10 μL from each dilution was spotted on the soft agar overlays of the host strains WA921 (*r-m-*), W3110 (*EcoK+*), WA2379 (*EcoA+*) and WA960 (*EcoB+*). Experiments were conducted in triplicate.

### 2.10. SDS-Page Analysis

For SDS-PAGE analysis, samples were prepared as described previously [6], with slight modification [17]. Briefly, approximately 2 × 10^10^ plaque-forming units of CsCl purified P1 were loaded per lane, normalized to band intensity of the tail sheath protein. SDS-PAGE was run on 4–20% Bis-Tris SDS-PAGE gels (Thermo Fisher Scientific, Waltham, MA, USA) with 5 uL PageRuler Unstained Broad Range Protein Ladder (Thermo Fisher Scientific) and stained with SYPRO Ruby (Thermo Fisher Scientific) following the manufacturer’s recommended protocol for maximum sensitivity. The gels were imaged with a Fotodyne gel imager.

### 2.11. Liquid Chromatography-Tandem Mass-Spectrometry of Purified P1 Virions

Samples were analyzed by LC-MS/MS using an Ultimate 3000 nano-LC system (Thermo) coupled to an Orbitrap Fusion tribrid mass spectrometer (Thermo, Waltham, MA, USA). Here, 1 µL of the sample was injected onto and separated by a 150 × 0.075 mm C18 column (Thermo Scientific Acclaim PepMap RSLC, 2 µm particle size) at a flow rate of 0.400 µL/minute. The total duration of the method was 60 min, with the gradient as follows: equilibration at 2% B (98% acetonitrile, 2% water, 0.1% formic acid), ramp to 45% B at 37 min, ramp to 90% B at 40 min and hold until 46 min, ramp down to 2% B at 47 min and hold at 2% B until the end of the run at 60 min. An eluent was introduced into the Fusion mass spectrometer by nano-ESI at a static voltage of 2450 V, with a transfer capillary temperature of 275 °C. Mass spectrometry data were acquired in positive mode at a resolution of 120,000 (at m/z 200) in the m/z range 400–1600. The RF lens was set to 60%. Maximum injection time was 100 ms. Scans were acquired in top speed mode with a cycle duration (survey scan plus as many dependent scans as possible) set to 3 s. Monoisotopic peak determination was used in peptide mode. The intensity threshold for precursors of interest was 5.0 × 10^3^. Charge states 1–6 were considered. Dynamic exclusion was set to 60 s with a mass tolerance of 10 ppm. MS/MS data were acquired by HCD at a fixed collision energy of 28% with a precursor ion isolation window of 1.6 m/z; fragments were detected in the ion trap at a rapid scan rate. Raw data were analyzed using Proteome Discoverer 2.1 (Thermo Scientific). MS/MS data were automatically extracted and matched against the P1 phage proteome database using the Sequest HT search engine. The generated list of detected proteins was further filtered to only include confident hits with a minimum of two unique peptides detected.

### 2.12. Bioinformatic Analyses

Protein sequences were searched against public databases by BLASTp [18] and HHpred [19] as appropriate. BLASTp searches were conducted against the NCBI nr database. HHpred searches were conducted against the PDB CIF70 (12 October 2021) and PFAM-A v35 databases at default settings. Results are reported at the matching database entry (as PDB or PFAM accession number) and the HHSearch percent probability score. In general, only matches with scores greater than 80% were considered credible, with most matches reported in this work having scores >95%.

### 2.13. Purification of Virions by CsCl Isopycnic Centrifugation

Purification of virions by CsCl isopycnic centrifugation was performed as previously described [6]. P1 or P1 mutants were induced in 1 L LB cultures as described above, concentrated by centrifugation, and centrifuged in a 0.75 g/L CsCl isopycnic gradient in a Beckman 70.1 Ti rotor for 24 h at 4 °C [17]. Phage bands were extracted with 18-gauge needles and dialyzed against 1 M NaCl SM buffer (1 M NaCl, 8 mM MgSO_4_, 50 mM Tris-HCl pH 7.5) for 24 h in Slide-A-Lyzer 3500 MWCO dialysis cassettes (Thermo Fisher Scientific), then dialyzed against fresh SM buffer for another 24 h. Phages were stored at 4 °C for further use.

### 2.14. Purification of Virion Sub-Assembly by CsCl Step-Gradient Centrifugation

P1 mutants were induced in 100 mL LB as described above. Here, 37 g of ammonium sulfate (Millipore Sigma, Burlington, MA, USA) was added to the lysate as it was shaking in ¼ increments every 15 min in a cold room. After the last addition, the lysate was allowed to shake for 1 h. The lysate was centrifuged at 8000× *g* for 30 min at 4 °C. The pellet was soaked in 5 mL SM buffer overnight. The lysate was filter sterilized using a 0.22 µM filter (Millipore Sigma). A shelf gradient of 1 mL 1.6 g/cc, 1 mL 1.4 g/cc, and 1 mL 1.2 g/cc CsCl, and 2 mL 20% sucrose was made in an Ultra-Clear™ centrifuge tube [9/16 × 3.5 inch (Beckman Coulter, Brea, CA, USA)]. The lysate was added to the top of the step gradient without disturbing the gradient. SM buffer was added using a serological pipette such that the tension of the SM buffer was flush with the opening of the tube. The tubes were loaded into a Beckman SW41 rotor and spun at 42,000 rpm for 2 h at room temperature. The bands were extracted with an 18-gauge needle and dialyzed as described above. Phages were stored at 4 °C for further use.

### 2.15. Complementation of P1 Δpmg Mutants

P1 knockouts were complemented *in trans* by induction of the mutant lysogen in a strain containing the expression vector of the complemented gene. Complementing genes were amplified by PCR from a P1 DNA template and cloned into pBAD24g at its XbaI and HindIII sites using standard molecular biology techniques [20]. Ligation products were transformed into competent *E. coli* 5-alpha cells (New England Biolabs) and selected by plating on LB agar amended with 20 µg/mL gentamicin. The plasmids were extracted as described above and were verified by sequencing before transforming the relevant *E. coli Δpmg* lysogen. Complemented P1 virions were generated by inducing the P1Δ*pmg* lysogen in conjunction with inducing the corresponding pmg complementing vector as follows. First, the transformed mutant lysogen was grown at 30 °C to an OD_550_ between 0.3 and 0.5. Next, L-arabinose (Millipore Sigma) was added to the culture to a final concentration of 1 mM and the incubation temperature was changed to 42 °C until the OD_550_ dropped below 0.2. Finally, 0.1% chloroform was added to the lysate, the lysate was centrifuged at 10,000× *g* at 4 °C for 30 min, and the supernatant was collected. The phage lysate was used to infect the non-restricting host *E. coli* WA921 by combining 100 µL of WA921 at OD_550_ between 0.3 and 0.6 with 100 µL of the phage lysate with 5 mM CaCl_2_ and allowed to incubate at room temperature for 30 min. The infection mixture was centrifuged for 1 min at 13,000 rpm and resuspended in 100 µL LB. The resuspension was plated on LB agar plates containing chloramphenicol and incubated at 30 °C overnight.

### 2.16. Edman Analysis

Parental P1 was produced and purified by cesium chloride isopycnic centrifugation as described above. Purified P1 virions were run on an SDS-PAGE gel and the band corresponding to the mature major capsid protein was prepared and submitted for analysis according to the protocol described by the UC Davis proteomics core for Edman sequencing analysis (Edman Sequencing Analysis Molecular Structural Facility, ucdavis.edu, accessed on 20 December 2021).

### 2.17. P1ΔdarB Transformation and pBAD24_N30-tetR-mCherry Induction

A lysogen of P1Δ*darB* [6] was grown in LB Cm-R Kan-R to an OD_550_ of between 0.3 and 0.5. One mL of the liquid culture was centrifuged at 13,000 rpm in a benchtop microcentrifuge (Eppendorf Centrifuge 5424) for 1 min and the supernatant was discarded. The bacterial pellet was resuspended and washed in 1 mL ice-cold ddH_2_O twice, followed by one wash in ice-cold 10% (*v*/*v*) glycerol in ddH_2_O and resuspension in 100 µL of ice-cold 10% glycerol in ddH_2_O containing 1 µL of either pBAD24_*darB*N30 or pBAD24_*darB*N9. The solution was transferred to an ice-cold 1 mm electroporation cuvette and electroporated using a Bio-Rad Micro-Pulser^TM^ following the manufacturer’s protocol. After electroporation, 900 µL of LB was added to the cuvette and the suspension was incubated at 30 °C for 1 h. The culture was centrifuged at 13,000 rpm, the supernatant was discarded, and the bacterial pellet was resuspended in 200 µL of LB. Then, 100 µL of the bacterial suspension was spread onto an LB Kan-R Amp-R agar plate, allowed to dry, and placed in a 30 °C incubator overnight. Transformant colonies were inoculated into LB Kan-R Amp-R, incubated overnight, subcultured 1:100 into 1 L LB Kan-R Amp-R and allowed to grow to an OD_550_ of 0.3–0.5. The cultures were then induced with 1 mM L-arabinose (Sigma-Aldrich), shifted to 42 °C, and incubated until the OD_550_ was below 0.2. The remaining cells were lysed by the addition of 0.1% (*v*/*v*) chloroform and the supernatants purified by isopycnic CsCl gradient centrifugation as described above.

### 2.18. Fluorescence Microscopy Imaging

Imaging was performed on a Nikon Eclipse Ti inverted epifluorescence microscope using a 100× objective (Plan Fluo, NA 1.40, oil immersion) with a 2.5× TV relay lens, within an incubator cage (InVivo Scientific, Salem, SC, USA) at 30 °C and images acquired using a cooled EMCCD (electron multiplying charge-couple device) camera (IXON 897, Andor, Belfast, UK). DAPI and mCherry images were collected using filter cubes for DAPI (Nikon no. 96310) and ET/mCH/TR (mCherry/Texas Red, Nikon no. 96365), respectively. Images were collected from 16 random fields and correlation of DAPI (400 ms exposure) and mCherry (500 ms exposure) foci was conducted manually in the NIS-Elements software (Nikon, Tokyo, Japan).

## 3. Results

### 3.1. Mass-Spectrometry Reveals the Proteome of the P1 Virion

Mass spectrometry is a sensitive application that can be used in a “bottom-up” approach to identify proteins within a sample [21]. This application has been used to identify structural proteins of multiple phages such as *Pseudomonas aeruginosa* phage O4 [22], *Salmonella* phage P22 [23], *Listeria monocytogenes* phage PSA [24], and coliphage T1 [25]. Although P1 is of significant historical importance, such modern approaches have not been applied to the P1 proteome. LC-MS/MS was conducted on trypsin digests of isopycnic CsCl gradient-purified P1 virions. DdrA is a known P1 structural protein [6] and yielded two unique peptides in this LC-MS/MS analysis, thus the value of two unique peptides was used as the cutoff for identifying a protein as a structural candidate.

Of the 119 open reading frames identified in P1 [10], 27 proteins meeting the two unique peptide cutoff were identified in our LC-MS/MS analysis of isopycnic CsCl purified P1 virions. Of these 27 proteins, nine of them produced a signal of two peptides each. However, four of these proteins (TciA, Cre, Dmt, and Ant1) have well-defined non-structural roles in the P1 life cycle [10], thus they were excluded from further consideration as virion structural components. Thus, based on this analysis, 23 proteins are attributed to the morphogenesis of the P1 virion (Table 1), including the six proteins known to comprise the virion-associated Dar system [6]. This number is in general agreement with a previous study based on visualization of protein bands in SDS-PAGE that suggested 28 proteins make up the P1 virion with 15 composing the head, 9 the tail, and 4 identified to be in the head or tail [26].

The total number of structural proteins detected in P1 is somewhat less than T4, which is composed of 37 structural proteins [11] but more than bacteriophage lambda, which is composed of 12 proteins [12]. Most of the identified structural proteins could be assigned a function and location in the virion based on previous studies [9,10] and additional bioinformatic analyses described below. Two novel proteins of previously unknown function, PmgC and PmgG, were also found to be virion-associated and are believed to play roles in tail or baseplate assembly, as discussed below. Much analysis of the P1 structure, particularly with respect to the tail and baseplate, will be discussed in the context of coliphage T4, a large myophage with extensive structural characterization [27] that also uses *E. coli* as its host.

#### 3.1.1. P1 Head Proteins Identified by LC-MS/MS

Nine proteins were assigned to the P1 head, including the major capsid gp23, portal (Prt), and the six proteins that comprise the P1 Dar system (Table 1). A capsid scaffolding protein has not been identified in P1, and it is possible that the scaffold of P1 is the N-terminal portion of the major capsid precursor protein as observed in phages such as HK97 [28] or T5 [29]. A weak signal (3 peptides, 9.7% coverage) of the predicted prohead protease was identified in the P1 virion. This finding has also been observed in the proteomic analysis of phage lambda [12], thus it is not clear if this plays a structural role or simply represents incomplete removal of this protein during morphogenesis. It appears that P1 also lacks a decoration protein(s), stabilization, or vertex proteins as seen in phages such as T4 [30].

**Figure 1:**
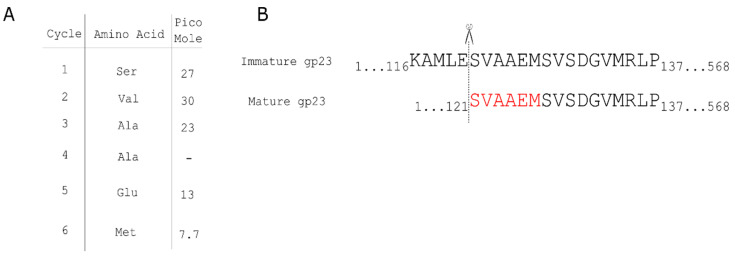
Edman degradation of P1 major capsid protein gp23. (**A**) Edman degradation reveals the first six amino acids of the N-terminus of gp23 purified from P1 virions. (**B**) The amino acids identified in the open reading frame of gp23, in which the first 120 residues are processed from the gp23 N terminus.

Proteolytic processing of the major capsid protein is a common feature of capsid formation in phages [31]. The transition between prohead I and prohead II of HK97 is characterized by the cleavage of a 104-residue polypeptide from the N-terminus of the major capsid protein by the phage-encoded protease gp4 [32]. The procapsid of phage T4 also undergoes proteolytic cleavage by the T4 protein gp21 [11]. In P1, processing of the major capsid protein gp23 has been presumed based on the discrepancy between the predicted length of the capsid gene product and the observed size of the mature P1 gp23 in SDS-PAGE gels. However, the processing site had not been determined. Edman degradation was employed to determine the primary sequence of the N-terminus of mature P1 gp23. The N-terminal residues of the mature P1 gp23 were determined to be Ser-Val-Ala-Ala-Glu-Met (Figure 1), indicating 120 residues from the N-terminus of gp23 are removed during capsid morphogenesis. The predicted P1 prohead protease Pro is the most likely candidate to carry out this processing [10]. DarA is also believed to undergo proteolytic processing [33]; however, a similar preparation of the mature DarA protein did not produce a clear consensus signal from N-terminal sequencing.

The capsid protein of HK97 is considered as a model in understanding the ancestry of myophage capsid evolution [34]. There may be parallels between the capsid assembly of P1 and HK97 as a two-sequence alignment in HHpred shows the secondary structure of P1 Pro and HK97 gp4 are conserved between amino acids 89 and 198 of the P1 sequence. It has been shown that linker insertion mutations in residues 130, 140, and 179 in gp4 of HK97 disrupt the activity of gp4 [35], and the conservation of this region in P1 would suggest a similar phenotype. HHpred alignment between the HK97 capsid protein gp5 and P1 gp23 revealed conservation in secondary structure between gp23 residues 307–469, respectively. This region includes the C-terminal portion of the gp5 E-loop and the betaC through betaF domains but lacks loop and alpha4. This suggests that HK97 may serve as a model for P1 head maturation.

#### 3.1.2. P1 Tail Proteins Identified by LC-MS/MS

As a myophage, the P1 tail is expected to be composed mainly of tail tube and sheath protein. The P1 tail sheath protein has been identified as gp22 based on previous work [10] and this is confirmed here with 51.6% peptide coverage (Table 1) and a strong HHpred match to the T4 tail sheath gp18 (Supplementary Table S4). The identity of the tail tube protein has been more difficult to confirm. Both Tub and BplB produced similar signals in the LC-MS/MS analysis (Table 1) and both have strong matches (>99.5%) to the T4 tail tube protein gp19 (5IV5_IB) as determined by HHpred. BLASTp alignment of Tub and BplB returns a short region of amino acid similarity (22 residues, 30% identity, E = 0.015) that suggests these proteins may be the result of a gene duplication event in the distant past. Alignment of these two proteins in HHpred shows strong secondary sequence conservation, and their similar relationships to T4 gp19 indicate that both proteins could plausibly function as the major tail tube component.

There has been a historical debate on the identity of the P1 tape measure protein, with both gp6 and Sit annotated with this function [10]. While gene *6* knockouts produced variable tail length in P1 [9], analysis by HHpred suggests it is a baseplate component, related to both the T4 gp27 central spike (P17172, 99%) and the phage Mu baseplate hub gp44 (1WRU_A, 99%). P1 gp6 is also too short to function as a tape measure protein: at 338 aa, gp6 could not determine the length of the 120 nm P1 tail based on the assumption of 1.5 angstroms length per residue in an alpha-helical protein [36]. At 1140 aa in length, Sit is a more appropriate size for the tail tape measure protein. Protein Sit contains a recognizable soluble lytic transglycosylase (SLT) domain (HHpred 5OHU_A, 99%) which spans residues ~750–900 of the Sit protein [37], and such SLT domains are a feature of some phage tail tape measure proteins [25,26]. Analysis of Sit for predicted secondary structure by Quick2D [19] shows that the N-terminal two-thirds of the protein are predominantly alpha-helical. This is consistent with tape measure protein structure, and, in addition, the length of the Sit alpha-helical region is nearly 750 residues, which is congruent with the length of the P1 tail [38].

Other P1 proteins detected by LC-MS/MS (Table 1) and identified by HHpred (Supplementary Table S4) to have a predicted tail function are gp24 and PmgC. The total number of proteins identified to be part of the tail assembly in P1 is similar to the number required for tail assembly in T4 [11]. HHpred analysis showed P1 protein gp24 to have strong similarity (94.6% probability) to the T4 tail terminator protein gp15 (Supplementary Table S4), indicating P1 gp24 is located at the top of the tail at the tail-head interface [39]. This analysis also identified PmgC as a possible head-tail joining component, with matches to the T7 gp11 tail adaptor. Direct two-sequence alignments in HHpred showed alignment (84% probability) of P1 PmgC to the T4 gp13 neck protein [39], indicating a role in head-tail connection.

#### 3.1.3. P1 Baseplate Proteins Identified by LC-MS/MS

The myophage baseplate is the nucleating site for tail assembly and is therefore assembled before the tail [11]. In the well-studied myophage T4, nearly 150 subunits form oligomeric components, which compose the six wedges that make up the T4 baseplate [11]. The wedges form around a central hub with the aid of trimeric proteins gp9 [40] and gp12 [41]. There are 15 proteins identified in the T4 baseplate including the tape measure protein [27]. While the assembly process of the P1 baseplate is not known, mass-spectrometry identified five proteins that are predicted to play a role in baseplate structure and have been assigned roles by bioinformatic analysis: S, R, BplA, PmgG, and gp6 (Table 1, Supplementary Table S4). The tape measure protein Sit, which is a component of both the baseplate and tail, is discussed below. P1 proteins R and BplA are baseplate wedge components orthologous to T4 gp8 and gp6, respectively, based on HHpred analysis (Supplementary Table S4). P1 gp6 is annotated as the baseplate hub orthologous to T4 gp27 as noted above, and PmgG is here annotated as the T4 gp48-like baseplate/tail junction. P1 S is the well-characterized tail fiber and specificity determinant responsible for host recognition [10].

### 3.2. Identification of Additional Morphogenetic Genes in P1

A previously published analysis of the P1 genome [10] annotated the functions of 22 proteins as *pmg* (putative morphogenetic function, 16 genes) or *upf* (unknown protein function, 6 genes). To better understand the role of these P1 genes, a collection of 29 single-gene knockouts were produced (Supplementary Table S3). Of these, 24 mutants had no observable plaquing defect and did not exhibit changes in antirestriction activity (Supplementary Table S3, Supplementary Figure S1). However, five of these single-gene knockouts (*pmgA*, *pmgB*, *pmgC*, *pmgG*, and *pmgR*) were unable to produce infectious virions under normal induction and plating conditions, indicating that these genes encode essential functions (Supplementary Table S3).

To confirm that defects in plaque formation were due to each specific gene deletion, we complemented each *pmg* knockout lysogen by cloning the cognate gene into pBAD24g and expressing the vector *in trans*; this complementation vector was also used to transform the *E. coli* host used in soft agar overlays. While plaque formation was rescued for P1Δ*pmgR* by this approach, plaque formation was not restored for the remaining four mutants. Phage plaque formation in soft agar overlays requires multiple cycles of phage infection, replication, and lysis to produce a visible plaque [42]. It is possible that gene dosage or expression issues of the *in trans* system reduced phage burst sizes and thus prevented the recovery of the plaquing phenotype. To confirm if *in trans* complemented P1Δ*pmg* knockouts were able to produce infectious virions, we moved to an experimental approach that would measure lysogen establishment as a marker for successful P1Δ*pmg* complementation (Table 2).

Each P1Δ*pmg* lysogen was induced in the presence or absence of its cognate complementing plasmid, and the lysates used to infect 100 µL aliquots of *E. coli* WA921. Lysogen formation was measured by plating infected WA921 on LB agar with and without 10 µg/mL chloramphenicol and to enumerate colony-forming units. By comparing the number of lysogens established between complemented P1Δ*pmg* lysates and their respective uncomplemented P1Δ*pmg*, *in trans* complementation of each P1Δ*pmg* background was measured. We believe this to be a sensitive assay as P1 virions establish lysogeny at a consistent probability of ~30% [43]. As shown in Table 2, lysates from all *in trans*-complemented P1Δ*pmg* mutants produced between 5 to nearly 500-fold more lysogens than the uncomplemented P1Δ*pmg* backgrounds, indicating the restoration of the parental phenotype with varying degrees of efficiency.

#### 3.2.1. Assigning Roles to the Newly Identified Essential Genes in P1

**Figure 2:**
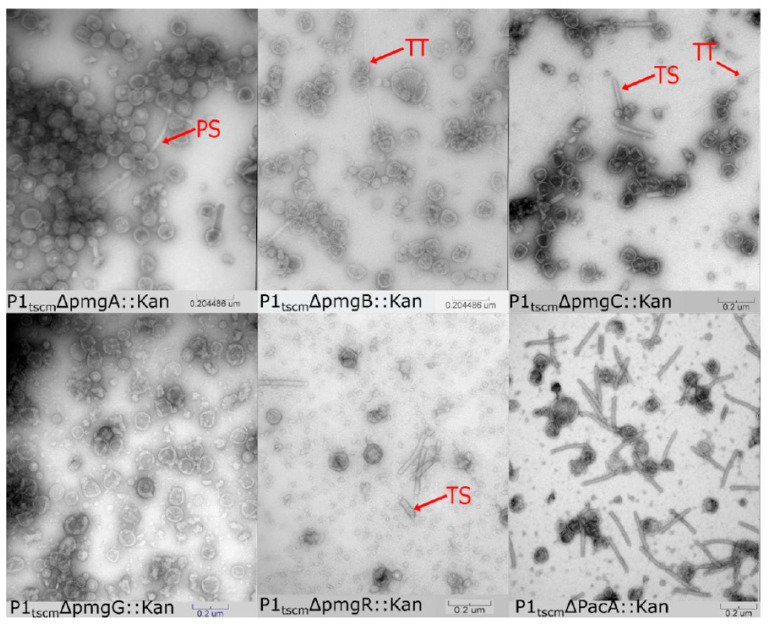
Transmission electron micrographs of CsCl step-gradient purified P1 morphogenic gene knockouts and parental P1. PS is polysheath, TT is tail tube, and TS is truncated sheath.

In tailed phages, tail and head assembly pathways are independent with the only interaction between the pathways occurring when the completed head and tail combine to form a functional virion [11]. Previous work on P1 predicted the roles of multiple morphogenetic genes by examination of P1 amber mutant lysates by transmission electron microscopy (TEM) [9]. Given the locations of the essential *pmg* genes in the P1 genome, we hypothesized that these genes play roles in phage morphogenesis. Virions of the five P1 *pmg* deletions and a *pacA* deletion were purified by cesium chloride step gradients and examined by TEM (Figure 2). The *pacA* mutant disables the P1 DNA packaging motor complex [44] and was used as a control for the purification of incomplete P1 virion components. In T4, defects in DNA packaging are known to result in phage heads stalled at an incomplete prohead state which lack packaged DNA and are unable to join with the assembled phage tails [45].

In the *pacA* knockout, phage proheads and complete phage tails were observed in roughly equal proportion (Figure 2), indicating that purification by cesium chloride step gradient can recover P1 virion components. Deletion of *pmgC* and *pmgR* produced lysates containing tails and incomplete heads but no complete P1 virions. This phenotype is consistent with the predicted function as a part of the head-tail connection complex described above. PmgR does not have a relationship detectable by HHpred to other proteins of known function, however, the similar phenotype in the *pmgR* and *pmgC* deletions suggests a role in head completion or head-tail joining. In contrast, deletions in *pmgA*, *pmgB*, and *pmgG* produced no observable complete tails but did produce possible aberrant polytube and poly-sheath structures as have been reported previously [9]. The heads of *pmgA*, *pmgB*, and *pmgG* knockouts are amorphous and resemble those of P1 mutants where tail assembly was perturbed [9], suggesting that completed P1 heads are unstable in purification if they remain incomplete due to lack of tail attachment. Analysis of PmgA and PmgG by HHpred suggest roles as baseplate components, with PmgA similar to the T4 gp25 inner baseplate wedge (5IW9_B, 99.4%) and PmgG similar to the T4 gp48 baseplate-tail tube junction protein (5IV5_R, 97.3%). PmgB is not related to proteins of known function, but the location of the *pmgB* gene immediately upstream of *sit*, the P1 tape measure gene, suggests a possible role as a tape measure chaperone [38].

Taken together, the data suggest that PmgA, PmgB, and PmgG play essential roles in tail morphogenesis while PmgC and PmgR play roles in head completion or head-tail attachment. Only PmgC and PmgG were detected in the virion proteomic analysis (Table 1). Failure to detect PmgB would be consistent with its hypothesized role as a tail chaperone and would not be expected to appear in the assembled virion [38]. Lack of LC-MS/MS signal for PmgA and PmgR may be due to their small size (13.2 kDa and 8.3 kDa, respectively), low copy number in the virion, and low numbers of peptides produced on trypsin digestion. While PmgA is predicted to act as a baseplate component, PmgR has no predicted function, and its deletion produces a phenotype of unjoined heads and tails. PmgR may play a role in head completion or head-tail joining but cannot be conclusively determined to be part of the virion at this time.

#### 3.2.2. Identification of Additional Structural Proteins

To identify additional structural components of the P1 virion, HHpred searches were conducted against all proteins of unknown function (annotated as *pmg*, *upf*, *uhr* or *upl*) and proteins implicated in morphogenesis in previous studies [10]. This analysis identified three additional P1 proteins, UpfC, gp5, and gp26, as having structural roles in P1 (Supplementary Table S4). UpfC shows a high-quality alignment with the T4 gp5.4 spike tip, which is attached to the C-terminal tip of the gp5 cell-puncturing tail needle [27,46]. The deletion of *upfC* did not result in any noticeable disruption to P1′s plaquing ability (Supplementary Table S3, Supplementary Figure S1), indicating this gene is dispensable in P1. In T4, gp5.4 also appears to not be essential for plaque formation (Petr Leiman, personal communication). P1 gp5 aligned in HHpred with the T4 gp5 tail needle/lysozyme, however, this alignment spanned only T4 gp5 residues 1–100 and 385–500, which corresponds roughly to the known N-terminal OB-fold domain and C-terminal beta-helix domain of T4 gp5, which forms the cell-puncturing needle structure [27,46]. This suggests that the P1 virion contains a cell-puncturing needle similar to that of T4, but this lacks the lysozyme activity associated with its T4 ortholog. Finally, P1 gp26 showed a good alignment with T4 gp53, which forms a part of the baseplate core in T4 [27] All of these proteins are relatively small (~10–20 kDa) and probably present in low copy number based on their T4 counterparts (UpfC, gp5 and gp26 present in 1, 3 and 6 copies per virion, respectively) [27]. This may be why these proteins were not identified in the LC-MS/MS analysis of P1 and it illustrates the limitations of this approach.

### 3.3. Localization of DarB into the Capsid

**Figure 3:**
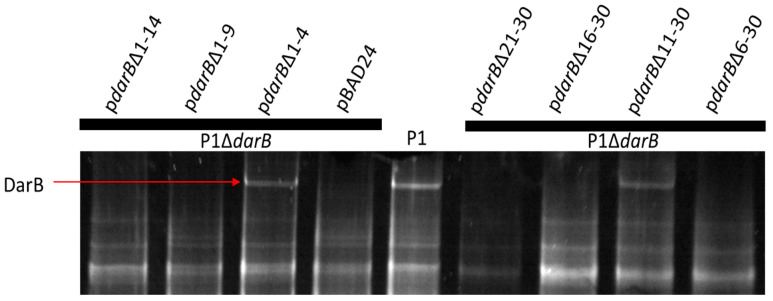
The ability of DarB truncations to localize to the P1 capsid. Truncations of DarB residues 1–4 and 11–30 are tolerated, which would suggest amino acids 6 through 10 are required to localize DarB to the P1 virion. However, deletion of residues 16–30 and 21–30 also inhibit DarB packaging, suggesting that other deletions may induce improper folding of the N-terminus that inhibits recruitment of DarB to the P1 virion.

P1 encodes the multi-component defense against the restriction (Dar) system that protects the P1 chromosome from host Type I restriction upon infection [5]. This system consists of six known proteins that are incorporated into the P1 virion in a stepwise process, in which the 250 kDa DarB protein is the last Dar component added to the capsid [6]. Phage T4 is understood to package internal proteins into its capsid, which are delivered to the host cell upon infection and play a role in defeating host restriction endonucleases [47]. Mullaney and Black [48] described a capsid targeting sequence (CTS) in the internal head protein IPIII of phage T4 that is sufficient to target foreign proteins to the T4 capsid while retaining their function. To determine if DarB is directed to the P1 capsid by a similar mechanism, truncation mutants at the DarB N-terminus were constructed (Figure 3).

**Figure 4:**
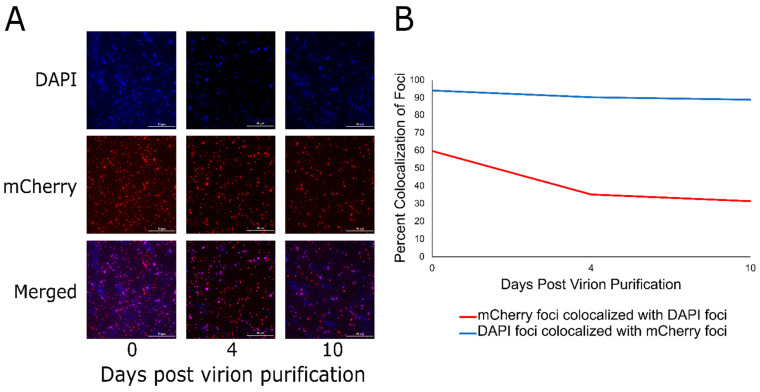
The 30 N-terminal residues of DarB are sufficient to target TetR-mCherry to the P1 capsid. (**A**) The simultaneous induction of the pBAD24_N30-tetR-mCherry expression vector and the P1Δ*darB* lysogen results in the localization of TetR-mCherry to the P1 capsid. Fluorescence imaging of CsCl-purified P1Δ*darB* + TetR-mCherry virions over days 0, 4, and 10 are shown using a DAPI filter, mCherry filter, and the two channels merged. (**B**) The line graph illustrates the colocalization of DAPI and mCherry foci over the 10 days post-purification of the P1Δ*darB* + TetR-mCherry virions. The red line indicates the percent colocalization of mCherry foci with DAPI foci, and the blue line indicates the percent colocalization of DAPI foci with mCherry foci. This indicates that most (~90%) particles with packaged DNA contain TetR-mCherry, but many particles containing TetR-mCherry do not contain DNA; this may be due to particle instability or premature DNA ejection from the virions during storage.

To further examine the DarB CTS, we wished to determine if this signal is sufficient to target a heterologous protein to the P1 capsid. To this end, the N-terminal 30 residues of DarB were fused to the N-terminus of the fluorescent reporter TetR-mCherry [16] and cloned into pBAD24 to generate the expression vector pBAD24_N30-tetR-mCherry. Next, a P1Δ*darB* lysogen was transformed with pBAD24_N30-tetR-mCherry. The P1Δ*darB* prophage and plasmid were simultaneously induced to produce P1Δ*darB* + TetR-mCherry virions. Finally, to visualize the localization of the TetR-mCherry to the P1 virion, virions were purified in CsCl isopycnic gradients and stained with the DNA intercalating dye 4′,6-diamidino-2-phenylindole (DAPI). As shown in Figure 4A, the DAPI and mCherry foci of cesium chloride isopycnic centrifugation purified P1Δ*darB* + TetR-mCherry virions are easily distinguishable in fluorescence microscopy. Fluorescence microscopy images were taken over a 10-day period and ~500 foci were counted in each DAPI and mCherry channel on each of days 0, 4, and 10. While DAPI foci and mCherry foci colocalize above 90% over the 10-day period (Figure 4B, blue line), mCherry foci colocalize with DAPI foci at nearly 60% on day 0, which declines to approximately 30% on day 10 (Figure 4B, red line). P1Δ*darB* was also induced in the presence of TetR-mCherry lacking the DarB CTS, and this construct produced P1 virions lacking fluorescent signal (Supplementary Figure S2), indicating the CTS is required for TetR-mCherry packaging in the P1 virion. This process was also conducted with the first nine residues of DarB fused to the N-terminus of TetR-mCherry, but the fusion construct did not target fluorescent signal to the P1 virion, indicating the complete CTS is greater than nine residues and less than 30 residues in length.

The exogenous packaging of TetR-mCherry to the P1 virion is similar in principle to the protein expression, packaging, and processing (PEPP) system of phage T4 developed by Mullaney et al. [49]. The T4 PEPP system has been shown to target diverse substrates to the T4 capsid via its N-terminal packaging signal, including HIV-1 protease, micrococcal endonuclease from *Staphylococcus aureus*, restriction endonuclease *Eco*RI, luciferase, human granulocyte colony-stimulating factor (GCSF), green fluorescent protein (GFP), and the 99 amino acid C-terminus of amyloid precursor protein (APP) [49]. The P1 capsid targeting sequence described here appears to serve an analogous function as the capsid localization signal described for the T4 PEPP system. The P1 system, however, has the advantage of being coupled to a much broader host range phage than T4; P1 has been shown to infect hosts as distantly related as *Myxococcus* [50] suggesting the P1 system could be employed to deliver proteins to a wide variety of bacterial targets.

We quantified the colocalization of DAPI foci and mCherry foci to be 90%, while the number of mCherry foci and DAPI foci are initially 60% on day zero and this declines to 30% at day 10. The drop in colocalization of mCherry foci and DAPI foci over the 10-day period is likely due to auto-ejection of the DNA during storage. The steady colocalization of DAPI foci and mCherry foci over the ten-day period is understandable as these are assumed to be virions that do not have their ejection mechanism disturbed during storage, thus the capsid harbors DNA and TetR-mCherry. It is reasonable to not expect 100% colocalization of DAPI foci and mCherry foci since there is a minimum amount of TetR-mCherry that needs to be packaged in the P1 virion to observe fluorescence. Previous work indicated that ~40 copies of DarB are packaged per virion in bulk measurements [6], but this number may vary widely between individual virions. Another possibility is that the TetR-mCherry protein encounters difficulty in folding while packaged into the P1 virion. Additionally, a band containing only P1Δ*darB* + TetR-mCherry tail-less heads was collected from the CsCl gradient and imaged by fluorescence microscopy on day 0 and day 10 (Supplementary Figure S3A). The purified P1Δ*darB* + TetR-mCherry heads display a 90% colocalization over the 10-day period (Supplementary Figure S3B), which suggests that the low rate of colocalization in the purified virions is due to an instability in the P1 DNA ejection mechanism mediated by the presence of the functional phage tail.

It is reasonable to suspect that Dar proteins share a common CTS, but BLAST analysis does not reveal a consensus sequence among the N-termini of Dar proteins. While the data from individual truncations suggest the CTS for DarB lies in residues 6–10, the first 30 amino acids are shown to be sufficient to direct the reporter TetR-mCherry to the P1 capsid. It is possible that the N-terminus of DarB requires proper tertiary structure to localize to the P1 capsid, as certain N-terminal truncations could compromise folding at the N-terminus and inhibit capsid targeting.

### 3.4. Discussion and Conclusions

**Figure 5:**
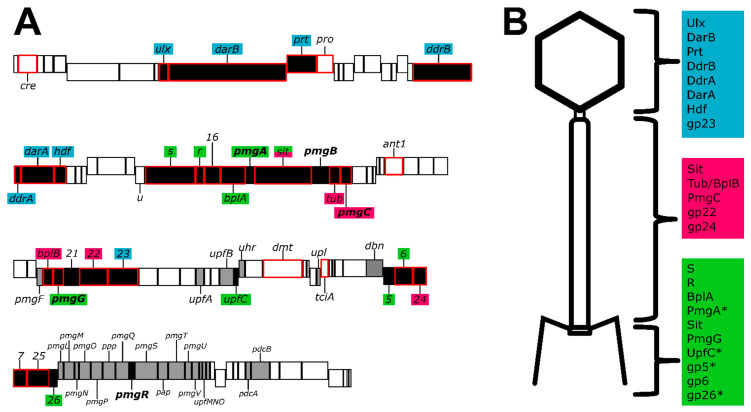
The P1 genome and composition of the virion. (**A**): Genome map of phage P1 summarizing P1 morphogenesis genes. The size of the boxes indicates the size of each ORF, with genes encoded on the plus and minus strands staggered up and down, respectively. Genes colored black are involved in phage morphogenesis, based on the work presented here or in previous studies [10]. Products of genes outlined in red were detected in LC-MS/MS above the two-peptide threshold. Genes with bold labels were determined to be essential for morphogenesis by construction of single-gene deletions. The deletion of gray genes produced no antirestriction phenotype and no apparent defect in plating, thus these genes are considered non-essential with unknown function. Gene labels are color-coded to indicate their predicted location in the virion (panel B), with blue indicating the head, magenta the tail, and green the baseplate. (**B**): A schematic of the P1 virion, partitioned into three components of the head, tail, and baseplate (including tail fibers). Structural proteins are assigned to each area based on their predicted roles; color coding corresponds to the coding of gene labels in panel A. Sit is listed in both the tail and baseplate as its role as tape measure protein makes it a part of both components. Proteins PmgA, UpfC, gp5, and gp26 are marked with an asterisk as these are predicted to be part of the virion based on bioinformatics but were not detected by LC-MS/MS. Gene *iddB* omitted for simplicity.

Bacteriophage genomes are known for their modular arrangements, with genes related to a specific function colocalized in the genome [12,51]. While many of the genes of P1 are bundled in such modules, multiple genes depart from this arrangement (Figure 5). For example, P1 gene *23* encoding the major capsid protein is located between gene *22*, encoding the tail sheath, and *parS*, encoding part of the P1 prophage partition system. The placement of P1 *23* is a departure from the placement of the major capsid gene in other model phages such as T4, lambda, and HK97, where the major capsid gene is closely associated with other genes related to capsid morphogenesis such as the scaffold, portal, or prohead protease [12,35,51]. Moreover, the 15 genes that have bioinformatic and LC-MS/MS evidence to support their role in tail or baseplate assembly (Figure 5) are scattered through the P1 genome. Genes numbered 30–41 are involved in baseplate, tail, and tail fiber morphogenesis and assembly, but genes encoding BplB, gp22, gp6, and gp24 are in positions 54, 57, 83, and 84, respectively. The placements for these genes depart from lambda genome architecture [12] but somewhat resemble the gene placement of T4 [51]. Further, genes 88 through 103 have been implicated as ‘head structure and processing’ [10], but our work (Supplementary Table S3) suggests that is not the case, as deletion of many of these genes does not appear to prevent the phage from making infectious virions.

A comparison of the proteins involved in baseplate formation in P1 and the paradigm phage T4 shows some significant disparities in their compositions. The T4 baseplate proper (excluding side tail fibers) is reported to contain 15 proteins, eight of which are proposed to form a conserved baseplate core [27]. In contrast, our analysis has been able to confidently assign only nine proteins to the P1 baseplate (Supplementary Table S5), suggesting the baseplate of P1 possesses a simpler structure than that of T4. Phage P1 has not been reported to possess short tail fibers analogous to T4 gp12, and such fibers are not evident in images showing P1 infection [52]. Thus, it is perhaps expected that orthologs of T4 gp9, gp10, gp11, and gp12 are absent in P1, as gp9 and gp10 are involved in the attachment of T4 gp12 to the baseplate [27]. Of the eight described core T4-like baseplate components, P1 possesses detectable orthologs of seven (Supplementary Table S5). This accounts for nearly the complete conserved inner baseplate core, with the exception of a clear ortholog of T4 gp7, which makes intimate contacts with gp6 in a 2:1 (gp6:gp7) heterotrimer and is conserved in myophage baseplates [27,53]. Searches of all annotated P1 proteins in HHpred were also unable to identify an ortholog of T4 gp7. A second baseplate-tail tube adaptor protein found in T4, gp54, is absent in P1, suggesting PmgG could fulfill this role alone. Electron micrographs showing the P1 baseplate (e.g., [52]) show a structure that is considerably more compact than the baseplate of T4, which would be consistent with a simplified baseplate structure in P1 relative to that of T4.

A T4 gp5-like needle ortholog was identified in P1 as gp5, but the P1 version of this protein appears to lack the lysozyme domain present in T4 gp5. This activity is likely supplied in P1 by the SLT domain found near the C-terminus of the predicted P1 tape measure protein Sit. Five additional P1 proteins were identified as virion-associated with a weak LC-MS/MS signal (gp7, gp16, and gp25), or were shown to be essential for virion morphogenesis (PmgR, PmgB). Protein similarity searches including HHpred were unable to identify possible roles for these proteins. These proteins may fulfill some of the functions associated with the “missing” T4 orthologs but are sufficiently diverged as to be no longer recognizable or be horizontal gene acquisitions. They may also play important non-structural roles in morphogenesis, such as acting as chaperones or cofactors analogous to proposed roles for T4 gp26 and gp51 [54].

In conclusion, while many of the essential genes of P1 have been elucidated using classical genetic approaches [9], approximately half of the genes in the P1 genome have no defined role. We have provided evidence that out of 29 genes with unknown function, five are essential for P1 morphogenesis while the remaining genes show no defect in plaquing, indicating that these functions are dispensable (Supplementary Figure S1). In addition, proteomic analysis of the virion validated previously identified P1 structural genes and identified two additional genes as being incorporated into the virion (Table 1). Combining new data with previously published work on P1 morphogenesis [9] and whole genome analysis [10] we are able to confidently assign locations to most of the P1 structural proteins identified by mass spectrometry. Taken together, this provides an updated genome annotation that can assign functions to multiple P1 proteins including baseplate, tail, and head-tail joining components (Figure 5). Updated bioinformatic analyses suggest that many P1 structural proteins have analogs in the well-studied myophage T4, including the capsid, portal, tail sheath, tail tube, nine baseplate components, and three tail completion or head-tail joining components. Also, like T4, P1 incorporates at least one protein, DarB, into the capsid via an N-terminal capsid targeting signal, and this signal can be exploited to direct a heterologous protein to the P1 capsid (Figure 4). Unlike T4, P1 does not appear to encode a separate scaffolding system for head assembly and may rather rely on a scaffold domain located at the N terminus of the major head protein as seen in phages such as T5 and HK97 [27,28]. Also, unlike T4, P1 appears to use a tail-associated muralytic enzyme that is fused to the end of the tape measure protein as seen in phages such as T5 [55] and TM4 [56] rather than a separate tail lysozyme analogous to T4 gp5 [30]. Remaining unresolved is the true identity of the P1 major tail tube subunit, as two candidate proteins, Tub and BplB, were identified as part of the virion and have matches to the T4 tail tube protein gp19.

We have developed a more complete picture of P1 assembly by identifying additional essential proteins required for morphogenesis, the capsid protein processing site, and a novel capsid targeting system that functions to package DarB and a heterologous protein to the P1 capsid. This system may make P1 an attractive agent for the delivery of fully formed proteins to a target cell and understanding the mechanisms of this system may allow the P1 Dar system to be modified to deliver novel cargo proteins.

## Figures and Tables

**Table 1:** Structural proteins identified by LC-MS/MS of purified *E. coli* phage P1 virions. Predicted functions are assigned based on previous studies or new bioinformatic analyses. Proteins are listed in the order of their open reading frame (ORF) number in the P1 genome.

Detected Protein	Predicted Function	Molecular Mass (kDa)	Number of Unique Peptides	Coverage (%)	Predicted Location of Protein	ORF Number	Source	GenBank ID
Ulx	Aids DarB localization	17	3	20.5	Head	9	[6]	2777418
DarB	Inhibition of EcoK and EcoB endonucleases	251.4	37	25.8	Head	10	[6]	2777481
Prt	Portal protein	62.7	11	37.7	Head	11		2777482
Pro	Prohead protease	36.8	3	9.7	Head ^†^	12		2777381
DdrB	Aids DarB addition to capsid	108.7	39	59.5	Head	22	[6]	2777413
DdrA	Antagonist of EcoA endonuclease	13	2	27.2	Head	23	[6]	2777414
DarA	Head size determinant and Dar system incorporation	69.4	27	67.4	Head	24	[6]	2777415
Hdf	Head size determinant and Dar system incorporation	22.1	7	39.9	Head	25	[6]	2777469
S	Tail fiber specificity	104.8	18	32.8	Baseplate	33	[10]	2777437
R	Baseplate wedge	15.9	3	26.3	Baseplate	34	This work	2777404
gp16	Baseplate structure *	31.3	2	11.5	Baseplate ^†,^*	35		2777405
BplA	Baseplate wedge	53.5	7	21.5	Baseplate	36	This work	2777406
Sit	Tape measure protein	120.6	23	27.8	Tail & Baseplate	38	This work	2777408
Tub	Tail tube ^a^	22.3	6	44.8	Tail	40	This work	2777479
PmgC	Tail adaptor	31.9	4	31	Tail	41	This work	2777480
BplB	Tail tube ^a^	18.7	7	33.1	Tail	54	This work	2777444
PmgG	Baseplate-tail tube junction	20.5	2	20.2	Baseplate	55	This work	2777384
gp22	Tail sheath protein	56.9	22	56.1	Tail	57	[10]	2777386
gp23	Major capsid protein	62.2	24	47.5	Head	58	[10]	2777387
gp6	Baseplate hub	37.2	4	16.8	Baseplate	83	This work	2777424
gp24	Tail terminator	28.9	5	22.2	Tail	84	This work	2777425
gp7	Tail Stability *	27.1	2	11.1	Tail ^†,^*	85	[10]	2777426
gp25	Tail Stability *	45.8	2	8.4	Tail ^†,^*	86	[10]	2777427

* Protein met the two-peptide threshold, but bioinformatic analysis does not support a clear structural role (see text and Supplementary Table S4); therefore, a putative role is assigned based on the annotation by Lobocka et al. [10]. ^†^ These proteins may be incorporated into the virion structure or serve as co-factors for virion assembly. ^a^ These proteins can both putatively function as tail tubes based on bioinformatic analysis (see text and Supplementary Table S4).

**Table 2:** Complementation of putative morphogenetic gene knockouts. Complementation of all mutants except for *pmgR* did not rescue the plaque-forming phenotype, thus complementation was observed by the restoration of the ability to produce infections virions that can establish lysogens in new hosts.

Lysate	Lysogens Established/mL Induced lysate	Fold Increase in Lysogen Establishment	Estimated Virions/mL in Induced Lysate
P1Δ*pmgA*	1.8 × 10^1^	-	6.0 × 10^2^
P1Δ*pmgA* + ppmgA	2.0 × 10^2^	11	6.7 × 10^3^
P1Δ*pmgB*	1.5 × 10^1^	-	5.0 × 10^2^
P1Δ*pmgB* + ppmgB	7.0 × 10^1^	4.7	2.3 × 10^2^
P1Δ*pmgC*	2.2 × 10^1^	-	7.3 × 10^2^
P1Δ*pmgC* + ppmgC	1.3 × 10^2^	6.1	4.4 × 10^3^
P1Δ*pmgG*	3.4 × 10^2^	-	1.1 × 10^4^
P1Δ*pmgG* + ppmgG	1.6 × 10^5^	460	5.3 × 10^6^
P1Δ*pmgR*	1.3 × 10^1^	-	4.3 × 10^2^
P1Δ*pmgR* + ppmgR	2.4 × 10^3^	180	8.0 × 10^4^
P1	3.0 × 10^5^	-	1.0 × 10^7^

## Data Availability

Not applicable.

## Supplementary Materials

The following are available online at https://www.mdpi.com/article/10.3390/v14040678/s1, Figure S1: P1 genes of unknown function are not required for antirestriction, Figure S2: P1ΔdarB+pBAD24_N30-mCherry and P1ΔdarB+pBAD24_mCherry virions were purified by side-by-side by CsCl isopycnic centrifugation and applied to slides for observation by fluorescence microscopy, Figure S3: P1ΔdarB capsids purified by cesium chloride isopycnic centrifugation, Table S1: Strains and plasmids used in this study, Table S2: Primers and synthetic DNA fragments, Table S3: EOPs of phage P1 single-gene knockouts, Table S4: Results of HHPred searches for P1 proteins identified as virion-associated by LC-MS/MS analysis or essential by genetic knockouts, Table S5: Baseplate components of coliphage T4 compared to baseplate.
