# Supplementary material for: New Insights into the Structure and Assembly of Bacteriophage P1
Source: Viruses. 2022 Mar 25;14(4):678. doi: 10.3390/v14040678 (PMC9024508; doi:10.3390/v14040678)
Supplement: Supplementary file 1 [file viruses-14-00678-s001.zip › viruses-1558289-supplementary.pdf]

<u>**Supplementary Material**</u>

Supplementary Table S1. Strains and plasmids used in this study.

**Strains, Phages, or Plasmids**	**Genotype or Relevant Characteristics**	**Reference/Source**
E. coli		
BW25113(pKD46)	F-, *Δ(araD-araB)567*, *ΔlacZ4787*(::rrnB-3), λ^{-}, *rph-1*, *Δ(rhaD-rhaB)568*, *hsdR514*, pKD46	The Coli Genetic Stock Center, Yale University (CGSC)
MG1655	*lacI*^{q1} *tonA*::Tn10	Lab stock
WA921	F-, *thr-1*, *leuB6*(Am), *fhuA21*, *lacY1 or Δ(cod-lacI)6*, *glnX44*(AS), λ^{-}, *metB1*, *thiE1*?, *hsdS3*	CGSC
W3110	F-, λ^{-}, *IN(rrnD-rrnE)1*, *rph-1*	CGSC
WA2379	F-, *leuB6*(Am), *fhuA21*, *lacY1 or Δ(cod-lacI)6*, *glnX44*(AS), λ^{-}, *metB1*, *thiE1*?, *deoB20*	CGSC
WA960	F-, *leuB6*(Am), *fhuA21*, *lacY1 or Δ(cod-lacI)6*, *glnX44*(AS), λ^{-}, *metB1*, *thiE1*?, *[r+B,m+B]*	CGSC
5-alpha competent	Used for its high efficiency transformation	New England Biolabs
**Phages**		
P1CM*clr*100	*Clr*100 ts; Chloramphenicol resistant	CGSC
P1Δ*darB*	In-frame deletion of *darB* in P1CM*clr*100	Piya et al., 2017
P1Δ*pmgA*	In-frame deletion of *pmgA* in P1CM*clr*100	This Work
P1Δ*pmgB*	In-frame deletion of *pmgB* in P1CM*clr*100	This Work
P1Δ*pmgC*	In-frame deletion of *pmgC* in P1CM*clr*100	This Work
P1Δ*pmgG*	In-frame deletion of *pmgG* in P1CM*clr*100	This Work
P1Δ*pmgR*	In-frame deletion of *pmgR* in P1CM*clr*100	This Work
P1Δ*pmgF*	In-frame deletion of *pmgF* in P1CM*clr*100	This Work
P1Δ*pmgL*	In-frame deletion of *pmgL* in P1CM*clr*100	This Work
P1Δ*pmgM*	In-frame deletion of *pmgM* in P1CM*clr*100	This Work
P1Δ*pmgN*	In-frame deletion of *pmgN* in P1CM*clr*100	This Work
P1Δ*pmgO*	In-frame deletion of *pmgO* in P1CM*clr*100	This Work
P1Δ*pmgP*	In-frame deletion of *pmgP* in P1CM*clr*100	This Work
P1Δ*pmgQ*	In-frame deletion of *pmgQ* in P1CM*clr*100	This Work
P1Δ*pmgS*	In-frame deletion of *pmgS* in P1CM*clr*100	This Work
P1Δ*pmgT*	In-frame deletion of *pmgT* in P1CM*clr*100	This Work
P1Δ*pmgU*	In-frame deletion of *pmgU* in P1CM*clr*100	This Work
P1Δ*pmgV*	In-frame deletion of *pmgV* in P1CM*clr*100	This Work
P1Δ*upfA*	In-frame deletion of *upfA* in P1CM*clr*100	This Work
P1Δ*upfB*	In-frame deletion of *upfB* in P1CM*clr*100	This Work
P1Δ*upfC*	In-frame deletion of *upfC* in P1CM*clr*100	This Work
P1Δ*upfM*	In-frame deletion of *upfM* in P1CM*clr*100	This Work

P1Δ*upfN*	In-frame deletion of *upfN* in P1CM*clr*100	This Work
P1Δ*upfO*	In-frame deletion of *upfO* in P1CM*clr*100	This Work
P1Δ*pdcA*	In-frame deletion of *pdcA* in P1CM*clr*100	This Work
P1Δ*pdcB*	In-frame deletion of *pdcB* in P1CM*clr*100	This Work
P1Δ*prt*	In-frame deletion of *prt* in P1CM*clr*100	This Work
P1Δ*pro*	In-frame deletion of *pro* in P1CM*clr*100	This Work
P1Δ*uhr*	In-frame deletion of *uhr* in P1CM*clr*100	This Work
P1Δ*upl*	In-frame deletion of *upl* in P1CM*clr*100	This Work
P1Δ*dbn*	In-frame deletion of *dbn* in P1CM*clr*100	This Work
P1Δ*ppp*	In-frame deletion of *ppp* in P1CM*clr*100	This Work
P1Δ*pap*	In-frame deletion of *pap* in P1CM*clr*100	This Work
**Plasmids**		
pBAD24	Empty cloning vector	Piya et al., 2017
pBAD24g	pBAD24 with gentamicin marker replacing ampicillin marker	This Work
ppmgA	P1 *pmgA* cloned into pBAD24g	This Work
ppmgB	P1 *pmgB* cloned into pBAD24g	This Work
ppmgC	P1 *pmgC* cloned into pBAD24g	This Work
ppmgG	P1 *pmgG* cloned into pBAD24g	This Work
ppmgR	P1 *pmgR* cloned into pBAD24g	This Work
pdarBΔ1-4	P1 *darB* missing the first 4 codons cloned into pBAD24	This Work
pdarBΔ1-9	P1 *darB* missing the first 9 codons cloned into pBAD24	This Work
pdarBΔ1-14	P1 *darB* missing the first 14 codons cloned into pBAD24	This Work
pdarBΔ1-19	P1 *darB* missing the first 19 codons cloned into pBAD24	This Work
pdarBΔ6-30	P1 *darB* missing codons 6-30 cloned into pBAD24	This Work
pdarBΔ11-30	P1 *darB* missing codons 11-30 cloned into pBAD24	This Work
pdarBΔ16-30	P1 *darB* missing codons 16-30 cloned into pBAD24	This Work
pdarBΔ21-30	P1 *darB* missing codons 21-30 cloned into pBAD24	
pFTSKi-tetR-mCherry	Template for PCR amplification of *tetR-mCherry*	(Shao et al., 2017) / Zeng Lab
pBAD24_N30	The first 30 amino acids of *darB* cloned into pBAD24	This Work

pBAD24_N9	The first 9 amino acids of *darB* cloned into pBAD24	This Work
pBAD24_N30-tetR-mCherry	mCherry fused to the first 30 amino acids of P1 *darB* cloned into pBAD24	This Work
pBAD24_N9-tetR-mCherry	mCherry fused to the first 9 amino acids of P1 *darB* cloned into pBAD24	
pBAD24_tetR-mCherry	pBAD24 expressing tetR-mCherry alone	
pMB838 cassette	source of gentamicin resistance	Addgene / Lab stock

Supplementary Table S2. Primers and synthetic DNA fragments

Forward 5'-3'	Reverse 3'-5'	Use
tcaattgatgatccgttgatgaat gactacgcgagagttgattgatgg ccgtgtaggctggagctgcttc	ctgtctttagtcggtgttgttt tactcatagcaccacgtcctgt gtgataatgggaattagccatg gtcc	Knockout *pmgA* from P1 genome
gcttcccttctgggctttagtttt tctctttgaattaaggagcaagga tggtgtaggctggagctgcttc	ggaaaaacgcataggattcagt gagcctttcagcgtcctgaatc ggtataatgggaattagccatg gtcc	Knockout *pmgB* from P1 genome
gtagaatcggttaacacaccagat tctacgaggtttcaatgacaccac gagtgtaggctggagctgcttc	aagcaacattaagccccatatc agccccctcacttcaacatgga tgagaaatgggaattagccatg gtcc	Knockout *pmgC* from P1 genome
ggaactccggcctttaacttgaat ggctcctatagcttatggggttta cagtgtaggctggagctgcttc	agtgttattgtgccccatataa aatccttttactggaacgcccg aacaatatgggaattagccatg gtcc	Knockout *pmgG* from P1 genome
atgtacactcatcacgttttttat tagagcaatctacaaggtgcacta tggtgtaggctggagctgcttc	ttgattggttttgtgctgaagt gatcatttcaaagttccgtatt agcttgatgggaattagccatg gtcc	Knockout *pmgR* from P1 genome
tttttgtgcttaaatcatgtcaa tatagcgaaattttgagcatatta tggtgtaggctggagctgcttc	gataattccgggagagcggttg ggtttttcatgcttttctgcg cagagaatgggaattagccatg gtcc	Knockout *pmgF* from P1 genome
aaatgtatcattctgcccttaagt aggttcttcacgaggaaacaaaat tggtgtaggctggagctgcttc	aaactcttcctcagtaaagcgc aacataatcagtcaaatcctgc cggtcgatgggaattagccatg gtcc	Knockout *pmgL* from P1 genome
gcaaaacagcgtaaatatggcatg cgaccggcaggatttgactgatta tggtgtaggctggagctgcttc	agcgcacatgcactaattaatt tattattttaagcagcatacaa ccacttatgggaattagccatg gtcc	Knockout *pmgM* from P1 genome
catgtgcgctctttgtggtagtgc actttaacatcgggagaataatcg tggtgtaggctggagctgcttc	agctgcaaattcagcctccagg cgagcattcatttcagcgatta cagggtatgggaattagccatg gtcc	Knockout *pmgN* from P1 genome

gctgaatttgcagctgagaatgaa cataccacccagggcgactaggac tggtgtaggctggagctgcttc	ctgcctctaaatttattcttgg gaaacattgaattgcactgctc ctacttatgggaattagccatg gtcc	Knockout *pmgO* from P1 genome
gcggagtgccgattatagcggtca atggggccatcgactggcttaacc gcgtgtaggctggagctgcttc	gtgggtgctatcatgtattctt cagccattctttaagagtcatc tgcggaatgggaattagccatg gtcc	Knockout *pmgP* from P1 genome
ggaaacctgacattgatgaaagtg aaaggtgatggtgcagcatgaaga ttgtgtaggctggagctgcttc	tttaaaaatatggaattattag agcaatattattctgattctcg ctcaaaatgggaattagccatg gtcc	Knockout *pmgQ* from P1 genome
atacttaggaaaaatgaccgaagc acaagctaatacggaactttgaaa tggtgtaggctggagctgcttc	caccagcattaaaagtgacact gtaactatcagcgaacgtaaat agtgccatgggaattagccatg gtcc	Knockout *pmgS* from P1 genome
tctctatcacttttggcggcatcg tcgcgcatgggtaagggggatgca tggtgtaggctggagctgcttc	aatgtccggatagttgccagcc tcgtaacccagggattagcact ccagctatgggaattagccatg gtcc	Knockout *pmgT* from P1 genome
ttattacgccagatcatcataaac aagccgagaaaagtgacctagaaa gggtgtaggctggagctgcttc	aggtgtttgatgtgatctgcaa ctctcatacttcaccctcgctt gtatcgatgggaattagccatg gtcc	Knockout *pmgU* from P1 genome
ttgccgttggtggatgctggcgat acaagcgagggtgaagtatgagag ttgtgtaggctggagctgcttc	ttctgtatgtgctggtgggtac ctgtagttcagctttcgttggc atttaaatgggaattagccatg gtcc	Knockout *pmgV* from P1 genome
gttaatgattacaaccgagctatt agcggtaactaaaagggattttta tggtgtaggctggagctgcttc	ccaacgtagccggttggaaggg agtcgtattattctacgctttc gatgagatgggaattagccatg gtcc	Knockout *upfA* from P1 genome
gagacggcctagttcaggtaagtt aggagatatcatgctggaaaaaga ctgtgtaggctggagctgcttc	tgaagaaaaattatcaatgaag tcctttgttactgtgccgcttt gtttaaatgggaattagccatg gtcc	Knockout *upfB* from P1 genome
aatctaagttaaaataacgaaaat cagagcaaatcattggtgatgacg tggtgtaggctggagctgcttc	cgtctccggctgcaacggtaga gccgcatgaaacaggatcacca acgcatatgggaattagccatg gtcc	Knockout *upfC* from P1 genome
gatgaggataatagccagaatctg gctaataacaggcgcatctaaaaa tggtgtaggctggagctgcttc	tgccgtaagctcacgttaacga ctttctttcaccgaatccaact atataaatgggaattagccatg gtcc	Knockout *upfM* from P1 genome
aaacccaacccttatatagttgga ttcggtgaaagaaagtcgttaacg tggtgtaggctggagctgcttc	acaaagttatgcacttgcaaga gggccattttctaaatattgtg atgtttatgggaattagccatg gtcc	Knockout *upfN* from P1 genome
gaaacatcacaatatttagaaaat ggccctcttgcaagtgcataactt tggtgtaggctggagctgcttc	tcccatccagaataattgagta acgactattatttaaccagcaa agtaacatgggaattagccatg gtcc	Knockout *upfO* from P1 genome

tctgtcgccgacacgttacgtaga ttgtatggttctgcggagtagatt aagtgtaggctggagctgcttc	acatcacacctttaatcactga ttgggctttatctgctgcccgg cattctatgggaattagccatg gtcc	Knockout *pdcA* from P1 genome
gaatgccgggcagcagataaagcc caatcagtgattaaaggtgtgatg tggtgtaggctggagctgcttc	taaagaaatagcaatacattag agcaattttatctaacactcga cgaatgatgggaattagccatg gtcc	Knockout *pdcB* from P1 genome
aaattccgttcaaacacgatgtga attattctaattaaggtgcaatct tggtgtaggctggagctgcttc	tcagtgaccgttttcaaaacat cagtcattatcgtttccctctt taaagaatgggaattagccatg gtcc	Knockout *prt* from P1 genome
aaatccttgaatcggtctttaaag agggaaacgataatgactgatgtt ttgtgtaggctggagctgcttc	tttggtggttatttaaacggat tgattgaattattaaacgtgat gatgctatgggaattagccatg gtcc	Knockout *pro* from P1 genome
accattcagccatcgcccttcaat gggcatttgtttggagtcgtcaga tggtgtaggctggagctgcttc	atagtaatgtttttccagtgct taaacatgttgtaacccttgaa tatcaaatgggaattagccatg gtcc	Knockout *uhr* from P1 genome
ttcaccgcaacggaaagagcattc ctggtggacctgtagattgggata tggtgtaggctggagctgcttc	tggtgaatgcacaggctgatgt gccgcaactacagtagtgcgcg ctttgcatgggaattagccatg gtcc	Knockout *upl* from P1 genome
cggtttgataactatgccggtgct gactggcaagaggattattaatgc aagtgtaggctggagctgcttc	ggcttttctgttatgacgggtt caattttttatccgttaccgcg cgacggatgggaattagccatg gtcc	Knockout *dbn* from P1 genome
ggtattccgcagatgactcttaaa gaatggctgaagaatacatgatag cagtgtaggctggagctgcttc	ccagagcgaagaactaaagcaa tcttcatgctgcaccatcacct ttcactatgggaattagccatg gtcc	Knockout *ppp* from P1 genome
ggtggagtgcgcccaccagcattt ttttcgtccaatgaggagggcatt tggtgtaggctggagctgcttc	gttttaaaaatcaagatttatt agagcaattattgttgatgaag aagcgcatgggaattagccatg gtcc	Knockout *pap* from P1 genome
atcggacgtcatgttacgcagcag caacgatg	atcggagctcttaggtggcggt acttgggtc	PCR amplify gentamicin resistance cassette from pMB838
atcggagctcctgtcagaccaagt ttactcatatatactttagattg	atcggacgtcactcttcctttt tcaatattattgaag	PCR amplify pBAD24 backbone
ggggatcctctagaataaaggagg tcagtacatggccaataataacga aattgatcc	aaaacagccaagctttcatagc accacgtcctg	clone *pmgA* into pBAD24g
ggggatcctctagaataaaggagg gatgctaatgcttttaccccttttt ccc	aaacagccaagctttcagcgtc ctgaatcgg	clone *pmgB* into pBAD24g
ggggatcctctagaataaaggagg gatgctaatgacaccacgacaatt actc	aaacagccaagctttcacttca acatggatgagaaaag	clone *pmgC* into pBAD24g
ggggatcctctagaataaaggagg gatgctaatggctcctatagctta tggg	aaacagccaagcttttactgga acgcccgaa	clone *pmgG* into pBAD24g

ggggatcctctagaataaaggagg gatgctaatgtggccattccgacg	aaacagccaagctttcaaagtt ccgtattagcttgtg	clone *pmgR* into pBAD24g
atcgtctagagtaataaaggaggt atcgagatgtctatgggggtgttt cgctg	atcgaagcttttatgcgtattg ttggatgacggc	Truncate the N termial region of DarB by amino acids 4
atcgtctagagtaataaaggaggt atcgagatgcgctgttcaagtgtc agcg	atcgaagcttttatgcgtattg ttggatgacggc	Truncate the N termial region of DarB by amino acids 9
atcgtctagagtaataaaggaggt atcgagatgagcgaaatattgaaa tacattagggc	atcgaagcttttatgcgtattg ttggatgacggc	Truncate the N termial region of DarB by amino acids 14
atcgtctagagtaataaaggaggt atcgagatgtacattagggcaata acatctcaccg	atcgaagcttttatgcgtattg ttggatgacggc	Truncate the N termial region of DarB by amino acids 19
attaaatacggcgtggaaaagg	agatagcttgttcatatcgatacct	Internal deletion of amino acids 6-30 of DarB
attaaatacggcgtggaaaagg	gcgaaacacccccataga	Internal deletion of amino acids 11-30 of DarB
attaaatacggcgtggaaaagg	gctgacacttgaacagcg	Internal deletion of amino acids 16-30 of DarB
attaaatacggcgtggaaaagg	gtatttcaatatttcgctgacacttg	Internal deletion of amino acids 21-30 of DarB
actctctactgtttctccataccc g	tgtatcaggctgaaaatcttct ctcatc	PCR amplify synthetic DNA fragments designed to introduce N-terminal DarB residues into pBAD24
atcgagaattcgtgtctagattag ataaaagtaaagtgattaacagcg	atcgaggtaccttacttgtaca gctcgtccatgc	Clone mCherry to pBAD24_*darB*N30
Synthetic DNA Fragments 5'-3'		
gacgctttttatcgcaactctctactgtttctccatacccgtttttt gggctagcaggaggtaatacaccatgaacaagctatctatgggggtgt ttcgctgttcaagtgtcagcgaaatattgaaatacattagggcaataa catctcaccgagcgccggaattcaccatggtacccggggatcctctag agtcgacctgcaggcatgcaagcttggctgttttggcggatgagagaa gattttcagcctgatacagattaaatc		Synthetic DNA fragment to introduce 30 N-terminal DarB residues into pBAD24

Supplementary Table S3. EOPs of phage P1 single-gene knockouts. Phage mutants were induced from lysogens in the restrictionless WA921 and plated to *E. coli* strains expressing *EcoA, EcoB* and *EcoK*. The restriction phenotype was compared to the parental P1 as described previously in Piya *et al.* 2017. Six phage mutants did not produce viable P1 phages upon induction, denoted as "PFU/mL <10^2". All other knockouts produced infectious virions and showed a restriction phenotype similar to that of the parental P1 (see Supplementary Figure S1).

Gene Knockout	Plating Phenotype
pacA	PFU/mL < 10^2
pmgA	PFU/mL < 10^2
pmgB	PFU/mL <10^2
pmgC	PFU/mL <10^2
pmgG	PFU/mL <10^2
pmgR	PFU/mL <10^2
upfA	normal restriction
upfB	normal restriction
upfC	normal restriction
uhr	normal restriction
upl	normal restriction
dbn	normal restriction
pmgL	normal restriction
pmgM	normal restriction
pmgN	normal restriction
pmgO	normal restriction
pmgP	normal restriction
ppp	normal restriction
pmgQ	normal restriction
pmgS	normal restriction
pap	normal restriction
pmgT	normal restriction
pmgU	normal restriction
pmgV	normal restriction
upfM	normal restriction
upfN	normal restriction
upfO	normal restriction

pdcA	normal restriction
pdcB	normal restriction

Supplementary Table S4. Results of HHPred searches for P1 proteins identified as virion-associated by LC-MS/MS analysis or essential by genetic knockouts. Proteins Gp7, Gp25 and Gp16 did not return high-quality matches in HHPred and their functions remain unknown.

	P1 protein	Match accession	Probability (%)	Function	PMID
**Proteins identified by LC-MS/MS**					
	**gp23**	**5VF3_L**	**100**	**Bacteriophage T4 isometric capsid**	**28893988**
	**Prt**	**3JA7_B**	**100**	**Portal protein gp20; VIRAL PROTEIN; 3.63A {Enterobacteria phage T4}**	**26144253**
	**Pro**	**5JBL_A**	**99.44**	**Prohead core protein protease; protease pentamer, phage T4, prohead, HYDROLASE; 1.943A {Enterobacteria phage T4}**	**27667692**
	**DdrB**	**ND**		**Antirestriction component**	**28509398**
	**DarA**	**ND**		**Antirestriction component**	**28509398**
	**DarB**	**ND**		**Antirestriction component**	**28509398**
	**Hdf**	**ND**		**Antirestriction component**	**28509398**

	DdrA	ND		Antirestriction component	28509398
	Ulx	ND		Antirestriction component	28509398
	gp22	3J2M_Y	100	Tail sheath protein Gp18; bacteriophage T4, phage tail terminator protein, phage sheath protein, VIRAL PROTEIN; 15.0A {E	23434847
	Tub	5IV5_IB	99.94	Tail tube protein gp19; T4, baseplate-tail tube complex, pre-attachment, bacteriophage, bacterial virus, hexagonal, memb	27193680
	gp6	1WTH_D	99.06	Baseplate structural protein Gp27; Triple-stranded beta-helix, OB fold, pseudohexamer, T4 tail lysozyme, HUB, gp5-gp27,	15701513
	gp7	None >50%			
	gp25	None >50%			

	Sit	5OHU_A	98.92	Soluble lytic murein transglycosylase; Lytic Transglycosylase, LYASE; HET: PO4; 2.2A {Pseudomonas aeruginosa}	29632171
	BplA	5HX2_D	100	Baseplate wedge protein gp6; T4, baseplate, complex, VIRAL PROTEIN; 3.8A	26929357
	S	5YVQ_A	99.45	Tail fiber protein S; bacteriophage, VIRAL PROTEIN; HET: GOL; 2.103A {Escherichia phage Mu}	31209305
	R	6HHK_C	98.72	Gp105; bacteriophage baseplate protein, VIRAL PROTEIN; HET: MSE; 2.38A {Listeria phage A511}	30606715
	gp24	4HUD_C	94.58	Tail connector protein Gp15; Bacteriophage T4, phage tail terminator protein, gp15, VIRAL PROTEIN; 2.7001A {Enterobacter	23434847
	gp16	None >50%			

	**BplB**	**5IV5_IB**	**99.79**	**Tail tube protein gp19; T4, baseplate-tail tube complex, pre-attachment, bacteriophage, bacterial virus, hexagonal, memb**	**27193680**
	**PmgC**	**7BOX_N**	**87.4**	**T7 gp11 tail adaptor**	**32266588**
	**PmgG**	**5IV5_r**	**97.3**	**T4 tail tube assembly protein gp48; baseplate-tail tube junction**	**27193680**
**Additional proteins identified as essential**					
	**PmgA**	**5IW9_B**	**99.4%**	**T4 gp25 inner baseplate wedge, initiator of sheath polymerization**	**27193680**
	**PmgB**	**None >50%**		**Tape measure chaperone?**	
	**PmgR**	**None >50%**			
**Other proteins with bioinformatically assigned structural roles**					

	**UpfC**	**4KU0_D**	**99.4%**	**T4 gp5.4 baseplate needle structure**	
	**gp5**	**1WTH_A**	**99.67%**	**T4 gp5 tail needle**	
	**gp26**	**5HX2_F**	**98.9%**	**T4 gp53 wegde component**	

Supplementary Table S5. Baseplate components of coliphage T4 compared to baseplate components identified in phage P1. Functions of P1 proteins were predicted using HHPred at default settings. Detection of the P1 protein by LC-MS/MS of purified P1 virions is also shown (see Table 1). Nine P1 proteins with orthologs to T4 baseplate components were identified, most of these are in the baseplate core. P1 proteins with no matches in HHPred are denoted with dashes (--). T4 proteins and their functions are adapted from Taylor et al. 2016 (PMID 7193680).

				HHPred result		
**T4 protein**		**T4 function**	**P1 ortholog**	**Structure/ domain**	**% Probability**	**Detected in LC-MS/MS**
**Baseplate core proteins**						
	gp5.4	Spike tip	UpfC	4KU0_D	99.4%	No
	gp5	Hub, needle, tail lysozyme	gp5	1WTH_A	99.67%	No
	gp6	Wedge	BplA	5HX2_D	100	Yes
	gp7	Wedge	--	--	--	--
	gp25	Wedge	PmgA	5IW9_B	99.4%	No
	gp27	Hub	gp6	1WTH_D	99.06	Yes
	gp48	Baseplate-tail tube junction	PmgG	5IV5_r	97.3	Yes
	gp53	Wedge	gp26	5HX2_F	98.9%	No
**Baseplate other components**						

	gp8	Wedge	R	6HHK_C	98.72	Yes
	gp29	Hub, tape measure	Sit	5OHU_A	98.92	Yes
	gp54	Baseplate-tail tube junction	--	--	--	--
**Baseplate tail fiber attachment**						
	gp9	Wedge, LTF attachment site	--	--	--	--
	gp10	Wedge, STF attachment	--	--	--	--
	gp11	Wedge, STF binding interface	--	--	--	--
	gp12	Baseplate outer rim, STF	--	--	--	--

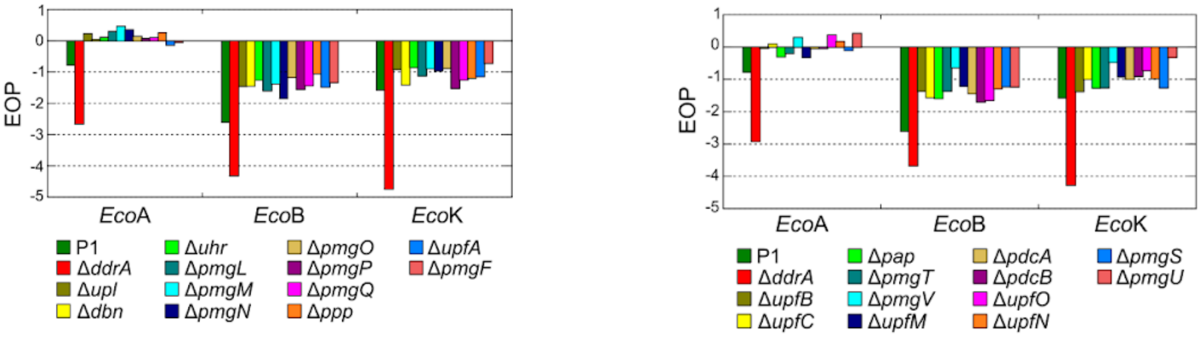

Supplementary Figure S1. P1 genes of unknown function are not required for antirestriction. Phage P1 or mutants were lysogenized into restriction-modification deficient E. coli strain WA921. The lysogens were thermally induced and tested for plating efficiency in E. coli strains with type I EcoA, EcoB or EcoK R-M systems. The EOP of parental P1, with functional antirestriction system is ~10^-1 , ~10^-3 or 10^-2 in strains with type I EcoA, EcoB or EcoK systems respectively, whereas the EOP of P1ΔddrA, with disrupted antirestriction system, is ~10^-3 , ~10^-4 or ~10^-4 -10^-5 in strains with type I EcoA, EcoB or EcoK systems respectively. The EOP of all other P1 mutants generated in this study appeared normal compared to the EOP of P1ΔddrA, suggesting that these genes do not play any roles in protecting P1 DNA from the type I R-M systems tested in this study.

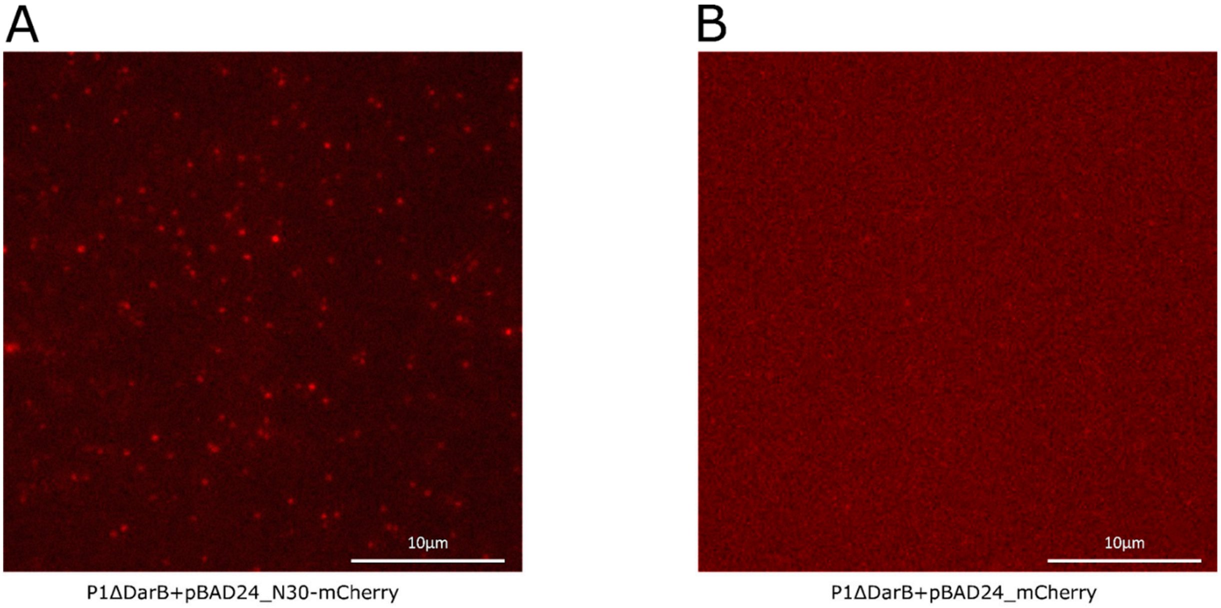

Supplementary Figure S2. P1Δ*darB*+pBAD24_N30-mCherry and P1Δ*darB*+pBAD24_mCherry virions were purified by side-by-side by CsCl isopycnic centrifugation and applied to slides for observation by fluorescence microscopy. (A) Fluorescence imaging of P1Δ*darB*+pBAD24_N30-mCherry shows fluorescent foci using the mCherry filter. (B) Fluorescence imaging of P1Δ*darB*+pBAD24_mCherry shows the absence of fluorescent foci using the same filter. Camera signal gain was set automatically for image capture, therefore the image background is amplified in (B), which appears dark under manual observation.

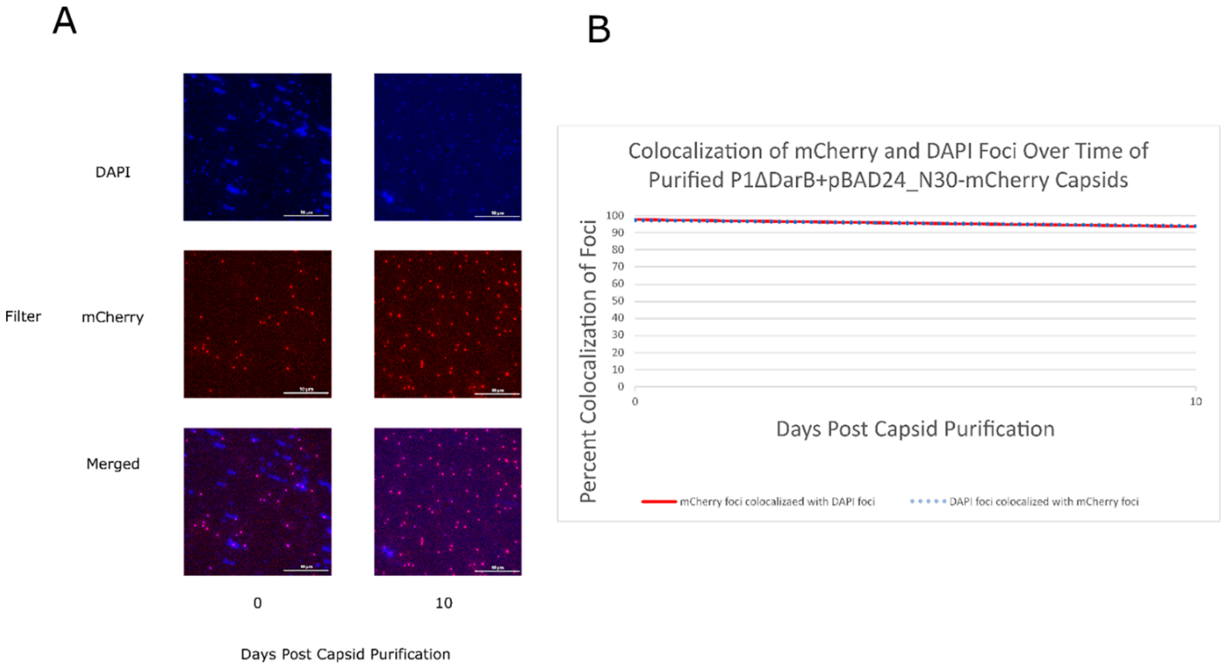

Supplementary Figure S3. P1Δ*darB* capsids purified by cesium chloride isopycnic centrifugation. (A) The simultaneous induction of the pBAD24_N30-mCherry expression vector and the P1Δ*darB* lysogen results in the localization of mCherry to the P1 capsid. Fluorescence imaging of cesium chloride isopycnic centrifugation P1Δ*darB*+pBAD24_N30-mCherry purified heads on days 0 and 10 are shown using a DAPI filter, mCherry filter, and the two filters merged. (B) The line graph illustrates the colocalization of DAPI and mCherry foci over the 10 days post cesium chloride isopycnic centrifugation of the P1Δ*darB*+pBAD24_N30-mCherry heads. The red line indicates the percent colocalization of mCherry foci and DAPI foci. Here, colocalization of foci occurs when an mCherry foci is observed first, then, secondly, the filter is switched to DAPI and the presence of a DAPI foci overlaps with the initial mCherry foci. The blue dotted line indicates the percent colocalization of DAPI foci and mCherry foci. Here, colocalization of foci occurs when a DAPI foci is observed first, then, secondly, the filter is switched to mCherry and the presence of an mCherry foci overlaps with the initial DAPI foci.
